# Three-dimensional, clinically rated posture data from people aged 10 to 69 years

**DOI:** 10.1016/j.dib.2024.110718

**Published:** 2024-07-06

**Authors:** Carlo Dindorf, Oliver Ludwig, Michael Fröhlich

**Affiliations:** Department of Sport Science, University of Kaiserslautern-Landau (RPTU), 67663 Kaiserslautern, Germany

**Keywords:** Normative data, Reference values, Flèche cervicale, Flèche lombaire, Lordosis, Kyphosis, Posture deviation, Machine learning

## Abstract

Weak posture is a widely recognized problem affecting individuals of all ages, that can lead to back pain, which is a significant socio-economic burden in civil societies. Posture assessment enables the early detection of postural deficiencies, thus enabling proactive interventions. Therefore, it is an important tool for promoting public health, not only in childhood and adolescence. This article provides posture data of 1,149 subjects aged 10 to 69 years measured by stereophotogrammetry. In addition to subject anthropometrics, raw sagittal posture parameters as well as calculated flèche cervicale (FC), flèche lombaire (FL), and kyphosis index (KI) and the respective values normalized to the trunk height (FC%, FL%, KI%) are provided. Further, based on the measurements and a visual inspection, biomedical experts made a classification of the presence of hyperkyphosis of the thoracic spine or hyperlordosis of the lumbar spine. In a second step, these assessments were algorithmically checked for label quality due to possible errors of the raters. Critical cases were reassessed by experts. In addition to the original ratings, these corrected labels are also reported. The data offers potential for data driven objective posture assessment and the development of diagnostic supportive machine learning applications.

Specifications Table

**Table:** 

Subject	Orthopaedics, Sports Medicine and Rehabilitation
Specific subject area	Posture deviations and normative data
Data format	Raw data with original and reassessed expert ratings for potential label errors
Type of data	Table (.csv file)
Data collection	Posture data were collected from healthy participants aged 10 to 69, excluding those with acute pain, spinal/musculoskeletal disorders, prior spinal surgery, leg length differences >5 mm, and vertigo. Weight/height were measured using a scale and stadiometer. Participants stood barefoot, in underwear, in front of a 3D scanner (Balance 4D, Paromed). Marker dots were placed at anatomical landmarks, and back contour was measured using coded light. Posture values (flèche cervicale, flèche lombaire, kyphosis index) were calculated and normalized to trunk height. Participants were classified for hyperkyphosis/hyperlordosis by biomedical specialists, with algorithmic checks for labeling quality. Critical cases were reassessed by experts.
Data source location	University of Kaiserslautern-Landau Department of Sports Science 67663 Kaiserslautern Germany
Data accessibility	Repository name: Mendeley Data Data identification number: 10.17632/5tv354mhs3.1 Direct URL to data: https://data.mendeley.com/datasets/5tv354mhs3/1
Related research article	Dindorf, C., Ludwig, O., Simon, S., Becker, S., & Fröhlich, M. (2023). Machine Learning and Explainable Artificial Intelligence Using Counterfactual Explanations for Evaluating Posture Parameters. *Bioengineering, 10*(5), 511.

## 1 Value of the Data

• By examining the dataset, researchers can **gain insights into age and gender-specific posture variations**. This information is essential for understanding how posture develops at different stages of life and how it differs between sexes. It can help in tailoring interventions and recommendations for specific demographic groups.
• Researchers can use this data to **refine objective, data driven diagnostic criteria** for hyperkyphosis of the thoracic spine and hyperlordosis of the lumbar spine and develop more accurate tools for identifying these conditions. This can lead to earlier intervention and better outcomes for affected individuals.
• Researchers and developers can utilize this dataset to **create applications for automated posture assessment**. Such applications can be valuable for individuals and healthcare professionals to monitor and improve their posture, potentially reducing the risk of associated health problems.
• The dataset provides a robust foundation for the **development and training of machine learning models**. These models can be designed to analyze posture data and make predictions or classifications related to posture quality. Such models can aid in early detection of posture-related issues, both in clinical and private settings, and offer personalized recommendations for improvement.

## 2 Background

Traditional methods of posture evaluation rely heavily on visual inspection by medical experts, a process prone to subjectivity and inconsistencies. These traditional methods exhibit moderate intra-rater reliability and weak inter-rater reliability [1], highlighting the need for more objective, data-driven approaches. Therefore, the primary motivation for compiling this dataset was to address the challenges and limitations in objectively assessing human posture, with a particular focus on the posture deficits hyperkyphosis and hyperlordosis.

Despite the established use of machine learning in other biomechanical applications, such as gait analysis and activity recognition, little prior research has explored its use in posture evaluation. Given the demonstrated potential of machine learning and deep learning in healthcare for analyzing complex, multivariate data and providing objective decision support, this gap presented an opportunity to extend the benefits of machine learning to posture analysis. Therefore, this dataset was compiled to explore the applicability of these technologies in posture evaluation for providing objective decision support and recommendations in the context of personalized medicine.

## 3 Data Description

**Fig. 1:**
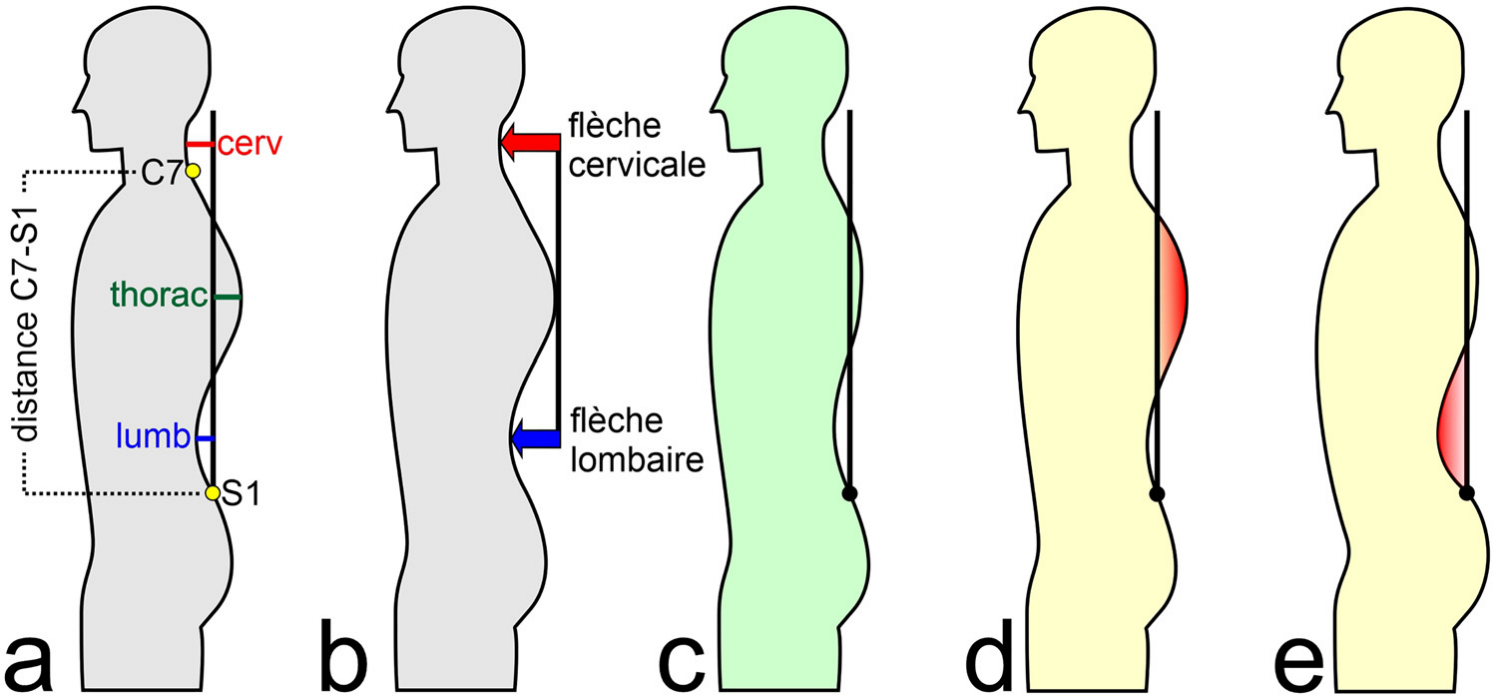
(a) Explanation of the analyzed posture parameters; (b) Calculated orthopedic posture parameters; (c) Normal posture, the plumb line begins at the first sacral vertebra; (d) Posture deviation hunchback (hyperkyphosis); (e) Posture deviation hollow back (hyperlordosis). Red areas mark the localization of the curvature deviation in (d) and (e).

This dataset [2] contains data of 1,149 participants (sex: 691 male, 458 female; age: 35.13 ± 15.91 years; weight: 73.86 ± 17.97 kg; height: 172.97 ± 10.17 cm). Table 1 gives an overview of the variables in the dataset of the article. Columns indexes 0 to 4 represent subject characteristics. The vertical distance between the 7th cervical and the 1st sacral as well as the horizontal distances of the 3 vertices to this perpendicular are found under columns indexes 5–8. For a better understanding of these parameters please refer to Fig. 1(a).

**Table 1:** Description of the data columns.

Column Index	Column Label	Description
0	age	in years
1	gender	male/female
2	height	body height in cm
3	weight	body weight in kg
4	BMI	weight/height^2^
5	distC7S1	vertical distance between the 7th cervical and the 1st sacral vertebra in mm
6	cerv	horizontal distance between the apex of the cervical lordosis and the perpendicular axis through the 1st sacral vertebra in mm
7	thorac	horizontal distance between the apex of the thoracal kyphosis and the perpendicular axis through the 1st sacral vertebra in mm
8	lumb	horizontal distance between the apex of the lumbar lordosis and the perpendicular axis through the 1st sacral vertebra in mm
9	KI	kyphosis index; (FC+FL)/2
10	FC	flèche cervicale; absolute value of difference between cerv and thorac
11	FL	flèche lombaire; absolute value of difference between lumb and thorac
12	KI%	KI x 100/ distC7S1
13	FC%	FC x 100/ distC7S1
14	FL%	FL x 100/ distC7S1
15	hyperkyphosis	postural deficit hyperkyphosis; 0 = not present, 1 = present
16	hyperlordosis	postural deficit hyperlordosis; 0 = not present, 1 = present
17	re_hyperkyphosis	reassessed postural deficit hyperkyphosis; 0 = not present, 1 = present
18	re_hyperlordosis	reassessed postural deficit hyperlordosis; 0 = not present, 1 = present

Based on these vertical and horizontal distances, the orthopedic parameters *fl*è*che cervicale, fl*è*che lombaire* and *kyphosis index* are calculated (column indexes 9–11). Flèche cervicale refers to the curvature of the cervical lordosis, which is the natural inward curvature of the neck. It is an essential part of the spine's alignment and helps maintain proper posture and head balance. Flèche lombaire relates to the curvature of lumbar lordosis, which is the natural inward curvature of the lower back. This curvature provides support for the body's weight and helps distribute it evenly. The kyphosis index measures the degree of forward curvature in the thoracic spine, which is the upper and mid-back region. An increased kyphosis index indicates an exaggerated rounding or hunching of the upper back, which can lead to poor posture and other related issues (see also Fig. 1; [3]). Additionally, these parameters were calculated as percentages of the trunk height (perpendicular distance between C7 and S1) and represented by column index 12–14. This standardization is performed to better take into account the individual variability in body size and shape.

The expert rating as well as the reassessed expert rating regarding the presence of the postural deficits *hyperlordosis* (hollow back) or *hyperkyphosis* (hunchback) are represented by the last four columns (index 15–18) [4]. Please note that this categorical data is unbalanced. Before reassessment, 729 participants exhibited hyperkyphosis while 420 did not, and 738 exhibited hyperlordosis while 411 did not. After reassessment, 725 participants exhibited hyperkyphosis while 424 did not, and 726 exhibited hyperlordosis while 423 did not. Hyperlordosis is an exaggerated inward curvature of the lower back (lumbar spine) that causes the pelvis to tilt forward and the abdomen to push forward. Hyperkyphosis is an excessive backward curvature of the upper back (thoracic spine), causing the upper back to round or hunch forward. Note, that both hyperlordosis and hyperkyphosis can be present in the same subject (see Fig. 1).

## 4 Experimental Design, Materials and Methods

The participants were volunteers from companies, associations, and various institutions. They received comprehensive information through verbal and written communication regarding the study's process and the relevant data protection guidelines. All participants provided written consent to participate. For individuals below the legal age, consent was additionally obtained from their legal guardians. Exclusion criteria encompassed acute pain, pre-existing spinal or musculoskeletal disorders, previous spinal surgery, leg length differences of more than 5 mm, and the presence of vertigo.

**Fig. 2:**
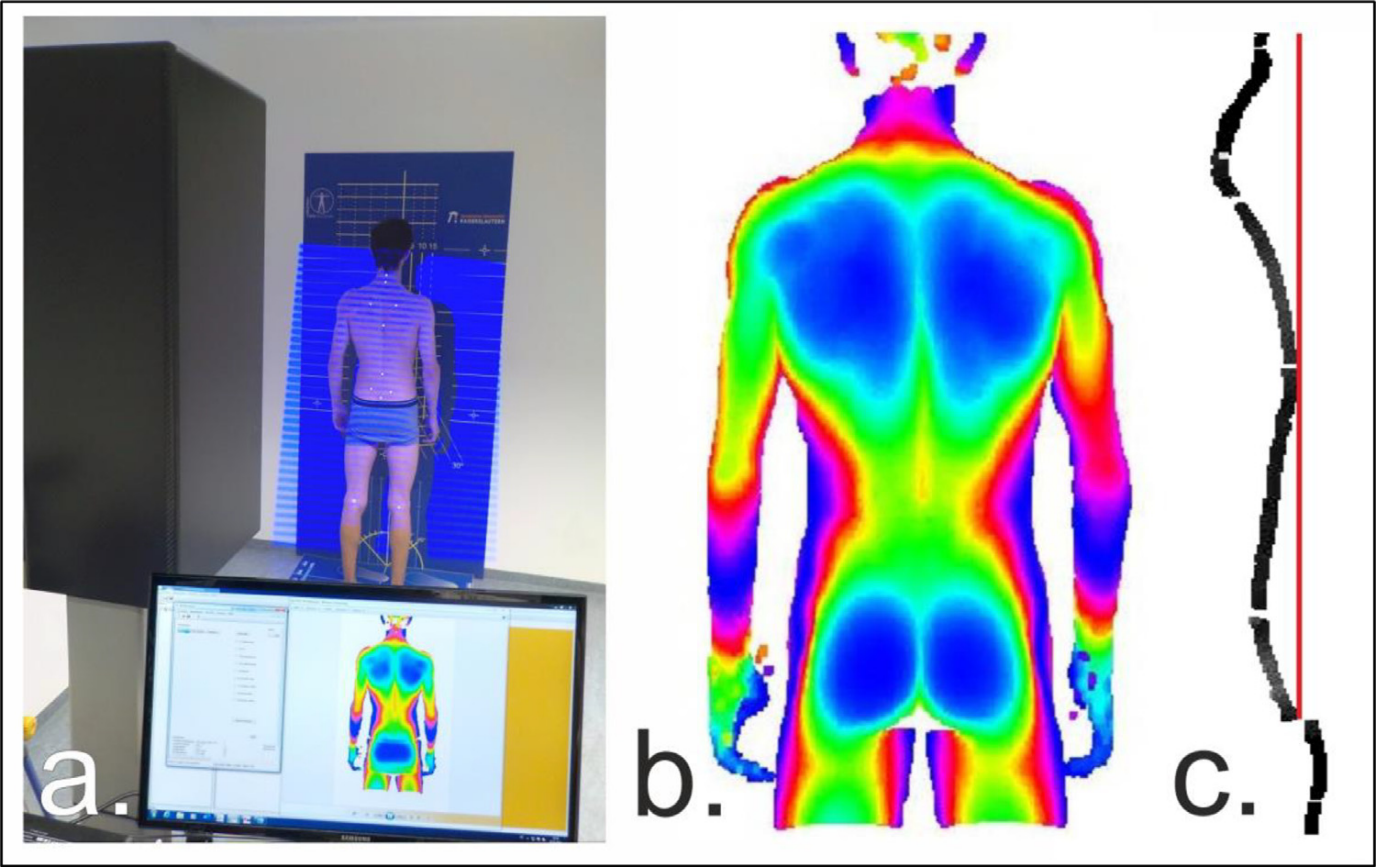
a. Test setup with 3D posture scanner during light strip projection; b. Colour-coded three-dimensional posture image; c. Computer-generated posture profile in sagittal plane as the basis for the reference values presented in this study. (For interpretation of the references to color in this figure legend, the reader is referred to the web version of this article.)

The examinations were conducted on-site at participating companies, institutions, or associations, within a designated room. The participants' body height was assessed using a stadiometer (Seca Stadiometer 213, Seca, Hamburg, Germany), weight was recorded using a scale (Robusta 814, Seca, Hamburg, Germany), and body mass index (BMI) was calculated accordingly. To measure posture, a portable 3D posture scanner (Balance 4D, Paromed bodybalance GmbH & Co KG, Neubeuern, Germany) was employed. This scanner utilizes a Vialux scanning unit (Vialux GmbH, Chemnitz, Germany), known for its proven precision and reliability [5]). The system is based on the projection of a dynamic light stripe pattern (LED light source, wavelength 460 ± 20 nm) onto the subject's back, achieving a spatial resolution of less than 1 mm. Prior studies have confirmed the validity of stereophotogrammetric methods for evaluating trunk shape [6,7]. During the measurements, participants stood barefoot (in underwear, men with bare upper body, women wore bras) in their natural postures approximately 2.30 m away from the device (see Fig. 2).

For the measurements, the investigator attached white adhesive dots (12 mm in diameter) to several anatomical landmarks. These included the spinous process of the seventh cervical vertebra (C7), the apex of cervical, thoracic, and lumbar spinal curves, the spinous process of the first sacral vertebra (S1), the posterior superior iliac spines (PSIS), and the angulus inferior of the scapulae. Each scan was conducted four times, and the resulting values were averaged. The system automatically recognized the anatomical landmarks, which, before further processing, were reviewed and verified by the investigator for precision. In case of deviations or points not recognized by the system, the measurement was repeated.

Following the measurements and a visual examination of the participants, four experienced biomedical specialists conducted a classification of the subjects based on the presence of hyperkyphosis in the thoracic spine or hyperlordosis in the lumbar spine. Each participant was assessed by only one of the four raters. All investigators possessed extensive expertise in posture analysis and adhered to consistent assessment criteria.

As studies demonstrated that experts tend to show diverging results regarding the rating of human posture [8], potentially wrong labels were flagged and re-evaluated using confident learning with the Python library cleanlab [9]. Cleanlab is a tool that aids in identifying and rectifying potentially mislabeled data points. Specifically, it assists in flagging instances where the model is confident about its predictions, but the labeled ground truth appears to be incorrect. The identified test data that raised concerns were subsequently reevaluated by competent specialists using a digital survey. Initially, two experts provided their evaluations for all the flagged subjects. In cases of conflicting assessments, a third expert was consulted, and the final class label was determined based on the majority vote. This procedure was essential for refining the dataset and enabling more accurate results in subsequent analyses. For a detailed description of the approach please refer to [10].

## Limitations

The present dataset has some limitations. First, it is important to note that we should refer to reference data rather than normative data due to dataset size and considering that the sample is not representative. Moreover, the participants were free of symptoms, which means that we cannot extend the findings to patients. It's well-established that individuals experiencing back pain often exhibit alterations in posture [10,11].

The measurement methodology employed has been well studied, and it has successfully met all established quality criteria [12,13]. However, the possibility of errors arising during the manual placement of individual marker points cannot be entirely ruled out. To mitigate this, extensive training was imparted to the involved researchers, each possessing substantial expertise in posture analysis, and they adhered rigorously to a standardized procedure. Despite these precautions, outliers may still be present in the dataset and may need to be addressed, as demonstrated in the related research article [4].

## Ethics Statement

The study was conducted according to the guidelines of the Declaration of Helsinki and was approved by the institutional ethics committees (Saarland University: UdS 15-6-08; RPTU: 23-57). All participants provided written consent to participate. For individuals below the legal age, consent was additionally secured from their legal guardians.

## CRediT authorship contribution statement

**Carlo Dindorf:** Conceptualization, Methodology, Software, Data curation, Writing – review & editing, Writing – original draft, Visualization, Investigation, Software, Validation, Writing – review & editing. **Oliver Ludwig:** Conceptualization, Methodology, Software, Data curation, Writing – review & editing, Writing – original draft, Software, Validation, Writing – review & editing. **Michael Fröhlich:** Supervision, Writing – review & editing.

## Data Availability

Posture data from people aged 10 to 69 years (Original data) (Mendeley Data).
